# Predicting lncRNA-protein interactions with bipartite graph embedding and deep graph neural networks

**DOI:** 10.3389/fgene.2023.1136672

**Published:** 2023-02-09

**Authors:** Yuzhou Ma, Han Zhang, Chen Jin, Chuanze Kang

**Affiliations:** ^1^ College of Artificial Intelligence, Nankai University, Tianjin, China; ^2^ College of Computer Science, Nankai University, Tianjin, China

**Keywords:** lncRNA-protein interaction, graph neural network, bipartite graph embedding, heterogeneous graph, link prediction

## Abstract

**Background:** Long non-coding RNAs (lncRNAs) play crucial roles in numerous biological processes. Investigation of the lncRNA-protein interaction contributes to discovering the undetected molecular functions of lncRNAs. In recent years, increasingly computational approaches have substituted the traditional time-consuming experiments utilized to crack the possible unknown associations. However, significant explorations of the heterogeneity in association prediction between lncRNA and protein are inadequate. It remains challenging to integrate the heterogeneity of lncRNA-protein interactions with graph neural network algorithms.

**Methods:** In this paper, we constructed a deep architecture based on GNN called BiHo-GNN, which is the first to integrate the properties of homogeneous with heterogeneous networks through bipartite graph embedding. Different from previous research, BiHo-GNN can capture the mechanism of molecular association by the data encoder of heterogeneous networks. Meanwhile, we design the process of mutual optimization between homogeneous and heterogeneous networks, which can promote the robustness of BiHo-GNN.

**Results:** We collected four datasets for predicting lncRNA-protein interaction and compared the performance of current prediction models on benchmarking dataset. In comparison with the performance of other models, BiHo-GNN outperforms existing bipartite graph-based methods.

**Conclusion:** Our BiHo-GNN integrates the bipartite graph with homogeneous graph networks. Based on this model structure, the lncRNA-protein interactions and potential associations can be predicted and discovered accurately.

## 1 Introduction

Long non-coding RNAs (LncRNAs) are RNAs with a length of no less than 200 nucleotides that do not encode proteins (Schaukowitch and Kim, 2014). Recently investigators have documented that lncRNAs play a critical role in various pathological and biological processes. Their misimpression can stimulate a series of lesions in humans, such as colon cancer (Pibouin et al., 2002), tumor initiation (Yang et al., 2014), nasopharyngeal carcinoma cell invasion (Wang et al., 2020a), and breast cancer (Wang et al., 2020b). For example, ncRNA miR-106b-5p assists metastasis by suppressing the key gene which links to breast cancer and activating Rho/ROCK1 pathway (Wang et al., 2020b). LncRNA ZNRD1-AS1 promotes the metastasis of nasopharyngeal carcinoma cells by regulating the miR-335–ROCK1 axis (Wang et al., 2020a). LncRNA LINC00337 promotes tumor angiogenesis, which can lead to colorectal cancer (Xu et al., 2021). Previous studies have shown that only a small fraction of the human genome is protein-coding genes (1.5%). In other words, the function of most human gene sequences that do not encode proteins is anonymous (Chen and Yan, 2013). Accordingly, the lncRNA-protein interactions are essential in analyzing the molecular function of lncRNAs.

Traditional methods can experimentally verify lncRNA-protein interactions. Previous experiments such as PAR-CLIP (Hafner et al., 2010) are time-consuming and cost-effective to predict, then computational approaches have been widely applied to lncRNA-protein interactions, which are based on machine learning and deep learning.

Graph neural network (GNN) is an extension method of a traditional neural network, which transforms the relationship between nodes into structured data and then completes forward propagation in graph domain (Scarselli et al., 2008). With the model iterations in GNN, existing research focuses on convolutions in graph data mining. Graph convolutional network is a variant of convolutional neural networks, which can operate directly on graph-structured data (Kipf and Welling, 2016). GraphSAGE (Hamilton et al., 2017) unifies the information of nodes through its neighbor node feature aggregation.

Link prediction using deep learning methods is commonly prescribed for disease-genes (Chen et al., 2018), miRNA-lncRNA (Huang et al., 2018) and many other fields. Previous study demonstrated that GNN had become the key instrument in link prediction (Zhang and Chen, 2018).

The following approaches achieve relevant results in predicting the interactions on lncRNA-protein. RPISeq method is a classifier for predicting LncRNA-protein interactions with two variants: Support Vector Machine (SVM) and Random Forest (Muppirala et al., 2011). NPI method integrated many advanced deep learning correlation models such as SEAL framework (Zhang and Chen, 2018) to this task (Shen et al., 2021). LPI-deepGBDT utilized gradient boosting decision trees for lncRNA–protein interaction identification (Zhou et al., 2021). LPIGAC implemented autoencoders on two graphs and trained these embedding collaboratively (Jin et al., 2021), however, their work was mainly based on the homogeneous graph, which led to the lack of the capability of heterogeneous features in the framework, including dependencies between heterogeneous nodes. The model results will be limited by some misjudgments, such as connecting homogenous nodes (protein-protein) and time-costing large-scale matrix calculations.

In recent years various methods based on graph embedding have been proposed. Li et al. (2015) proposed a heterogeneous network based on the protein-protein interaction. LPLNP was designed based on linear neighborhood propagation, which transfers the graph similarity into the network embedding (Zhang et al., 2018). LncPNet was proposed based on embedding the heterogenous network to learn the low-dimensional potential node representations (Zhao et al., 2022).

Major of the biomedical interaction graph is not homogeneous. For instance, lncRNA-protein, disease-genes, hence bipartite graph embedding is fundamental to predict the potential edge in the bipartite graph. Before bipartite embedding was proposed, many studies contributed to the work of homogeneous graph embedding (Cui et al., 2018; Cai et al., 2018). Although these methods work well, they are not suitable for embedding the construction of bipartite graphs. To remedy the problem, increasing explorations on heterogeneous graphs have been proposed. The reconstruction-based method with graph convolutional matrix completion works pretty well on standard collaborative filtering benchmarks (Berg et al., 2017).

The structure of the bipartite graph network has been iterated many times. Metapath2vec applied scalable node representation in heterogeneous networks (Dong et al., 2017). BiNE proposed a random walk generator to generate representation vectors and also combined explicit relations and implicit relations (Gao et al., 2020). BiRank proposed a method to integrate bipartite graph structure and node representation (He et al., 2016). BiGl integrated the embedding of two node types into local-global representation, which also proposed the bipartite embedding applied to deep learning (Cao et al., 2021).

Gilmer captured node representations by using the features of neighboring nodes to train the neural network (Gilmer et al., 2017). DMGI (Park et al., 2020) utilized the infomax objective to heterogeneous graphs. It splits the heterogeneous graph into homogeneous graphs and applies the infomax objective to this task.

In this study, we integrate homogeneous networks and heterogeneous networks to construct mutual optimization model through bipartite graph embedding. The heterogeneous features combine the association information to obtain the bipartite graph features of each node. The representations are input into the homogeneous network established based on GraphSAGE and matched with interaction to update the bipartite features. Finally, the bipartite embedding is input into the logistic regression classifier to calculate the link categories. In summary, the main advantages of BiHo-GNN are as follows:

1. BiHo-GNN can capture the feature of the lncRNA-protein interactions and distinguish the disparate nodes, which can lower the negative effect of lncRNA-protein homogenization on link prediction.
2. BiHo-GNN combines the advantages of heterogeneous and homogeneous networks, which uses the heterogeneous network to generate bipartite graph features. The bipartite embedding is a feature composed of two types of node prototype representations.
3. Our homogeneous network based on GraphSAGE can iterate bipartite embedding from heterogeneous network to form a feedback process.

## 2 Materials and methods

### 2.1 Datasets

There are four datasets collected in this study. These datasets are NPInter2.0 (Yuan et al., 2014), NPInter3.0_H (Hao et al., 2016), NPInter3.0_M (Hao et al., 2016), RPI2241 (Muppirala et al., 2011).

NPInter 2.0 database includes 10,412 experimentally demonstrated functional lncRNA-protein interactions, containing 4,636 RNAs and 449 proteins, which were extracted from the UniProt database (UniProt Consortium, 2014) and the NONCODE database (Bu et al., 2012). NPInter 3.0 is an upgraded dataset of ncRNA-sequence interactions. We only use two pieces of data for processing. NPInter3.0_H is from the *homo sapiens* specie part, composed of 7,317 lncRNA–protein interaction pairs, 1874 RNAs, and 118 proteins. NPInter3.0_M is the musculus species subset of NPInter 3.0, involving 1847 experimentally verified lncRNA–protein interactions. These interactions contain 1939 RNAs and 60 proteins. RPI2241 and the above datasets differ in data acquisition. RPI2241 is acquired based on 3D atom coordinates and algorithm inference (Lewis et al., 2010), containing 2,241 interactions, 838 RNAs, and 2040 proteins. These four datasets have an exact number of each type of node. At the same time, the bipartite embedding model in data preprocessing may lead to filtering out a few low-frequency nodes. The specific number of each item is shown in Table 1. Since these four datasets only marked positive samples, we randomly selected negative samples with the same number of positive samples in the data sets that have not been verified to be associated.

**TABLE 1:** Introduction of four datasets used in this paper.

Datasets	Species	Interactions	RNAs	Proteins
NPInter2.0	—	10,412	4,636	449
NPInter3.0_H	*Homo sapiens*	7,317	1874	118
NPInter3.0_M	*Mus musculus*	1847	1939	60
RPI2241	—	2,241	842	2,043

### 2.2 Background

Let *G* = (*L*, *P*, *E*) be the bipartite graph, where *L* and *P* are the set of RNAs and proteins, with *E* is the edges between RNA and protein sets. It is obvious that RNA and protein nodes are heterogeneous. Representation vectors are instrumental in their forward propagation in graph neural networks. For this bipartite graph with vertex sets *L* = {*l*_1_, …, *l*_*r*_}, *P* = {*p*_1_, …, *p*_*s*_}, where r and s denote the number of lncRNAs and proteins. Edges *E* ⊆ *L* × *P*, bi-adjacency matrix *A* ∈ {0,1}^*r*×*s*^, where *A*_*i*,*j*_ = 1 when the RNA node *l*_*i*_ ∈ *L* and the protein node *p*_*j*_ ∈ *P* interact, and *A*_*i*,*j*_ = 0 when no interaction occurs. Bipartite graph embedding maps graph data into a feature matrix based on a sample Bi-GNN by mutual iteration between molecule nodes.

### 2.3 Bipartite graph embedding

The bipartite graph embedding, which was proposed by Cao (Cao et al., 2021). Inspired by this work, we design the feature of lncRNA and protein nodes. In this part, we use Bi-GNN as the bipartite graph encoder to generate the molecular node representations and take these representations into the following network frame. For clarification of the notations, we use *l*_*i*_ and *p*_*j*_ to stand for the representation of RNAs and proteins node, respectively.

**FIGURE 1:**
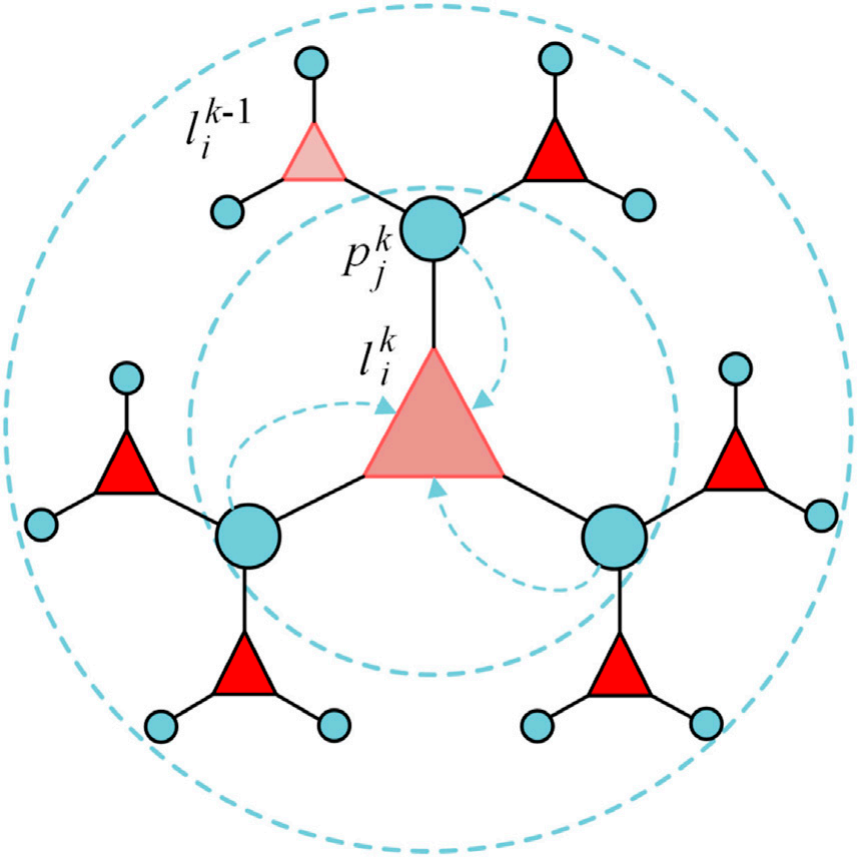
The forward propagation process of node from $l_{i}^{k-1}$ to $l_{i}^{k}$ .

The key problem in the prediction of lncRNA-protein interaction is the utilization of neighbor nodes attribution and utilize the homogeneity of two molecular nodes efficiently. Bipartite graph encoder can learn each node feature from two-hop neighbors interaction. Taking $l_{i}^{k-1}$ for example as illustration shown in Figure 1, each node forward propagation in *k*-th layer aggregates the embedding of two-hop neighbor nodes. The propagation of $l_{i}^{k}$ , $p_{j}^{k}$ is represented $l_{i}^{k-1}$ *via* a Bi-GNN encoder:

$$p_{j}^{k}=ReLU\left(\left.Linear\left(\hat{A}l_{i}^{k-1}W^{k-1}:l_{i}\in L\right\}\right)\right)$$ (1)

Protein nodes $p_{j}^{k}$ are represented by upper RNA nodes $l_{i}^{k-1}$ , $l_{i}^{k}$ can be obtained by the Bi-GNN encoder based on $p_{j}^{k-1}$ .

$${\hat{l}}_{i}^{k}=ReLU\left(\left.Linear\left(\hat{A}p_{j}^{k-1}W^{k-1}:p_{j}\in P\right\}\right)\right)$$ (2)

where $\hat{A}=D^{-1/2}\left(A+I_{n}\right)D^{1/2}$ , *A* is the adjacency matrix of the bipartite graph, *D* is the diagonal degree matrix of *A* + *I*_*n*_, and *W* denotes the weights of the GNN encoder. The final RNA node embedding $l_{n}^{k}$ is the cascading matrix of ${\hat{l}}_{n}^{k}$ and $l_{n}^{k-1}$ as follows:

$$l_{n}^{k}=Linear\left(\left[{\hat{l}}_{n}^{k}\;l_{n}^{k-1}\right]\right)$$ (3)

### 2.4 Network structure

Depending on the generated bipartite node embedding, in this section, we proposed a homogeneous network to learn RNA and protein embedding, which can capture the homogenous properties of bipartite embedding.

In homogenous network, both RNA and protein share the same node type and node representation. We integrate two types of nodes embedding as the new input $H\in R^{\left(r+s\right)\times d}$ of the homogenous network *via* a simple composition operation:

$$\begin{array}{c} H={\left[L\;P\right]}^{T} \end{array}$$ (4)

For each node type, we construct a bipartite node feature matrix under the interaction order, and the new node attribution *X* can be decoded with *H*_*l*_ and *H*_*p*_.

$$\begin{array}{c} X=H_{l}⊙H_{p} \end{array}$$ (5)

where *H*_*l*_ and *H*_*p*_ are the node feature order of RNA and protein.

In this paper, we applied GraphSAGE (Hamilton et al., 2017) to aggregate information from the neighborhood representation, which can extract the bipartite embedding for previously neglected nodes, and we add three rectified linear layers as the final feature to the softmax function.

$$Z=X\cdot \Theta^{\left(0\right)}+AGG\left(X_{q}:x_{q}\in N\left(x\right)\right)\cdot \Theta^{\left(1\right)}$$ (6)

where Θ denotes the weights matrix and N(x) denotes the *q*-hop neighbors of *x*.

### 2.5 Model training

From Eq. 4, we can obtain the lncRNA-protein prototype representation *H*, Bi-GNN encoder training loss function $L_{b}$ is defined as:

$$L_{b}=-\frac{1}{\left|E|+|E^{\prime }\right|}\overset{\left|E\right|}{\underset{i=1}{\sum }}\overset{\left|E^{\prime }\right|}{\underset{i=1}{\sum }}\left(y_{i}\left[\log\left(\phi \left(h_{i}\right)\right)\right]+y_{i}^{\prime }\left[\log\left(1-\phi \left(h_{i}^{\prime }\right)\right)\right]\right)$$ (7)

where *y*_*i*_ is the label of interaction and $y_{i}^{\prime }$ is the set of negative lncRNA-protein pairs. *ϕ* is a temporary classifier involved by a fully connected layer and a sigmoid function.

Through the optimization of node feature *H* in reconstruction loss $L_{r}$ , bipartite Graph embedding can be optimized in homogeneous networks.

$$L_{r}=-\overset{\left|E\right|}{\underset{i=1}{\sum }}y_{i}logx_{i}$$ (8)

**FIGURE 2:**
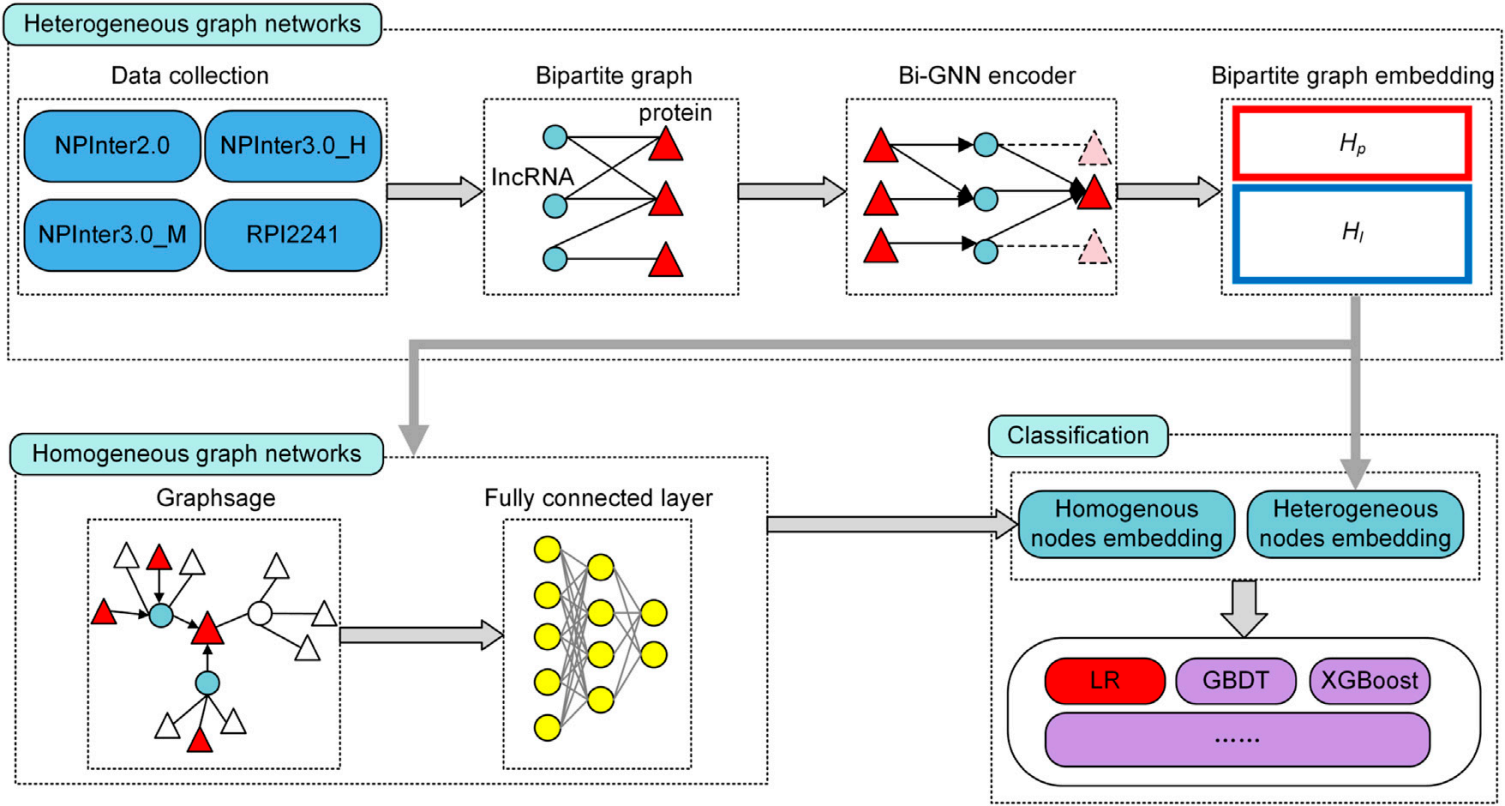
The structure of BiHo-GNN.

The final loss function L is composed of above two sections:

$$L=\alpha L_{b}+\left(1-\alpha \right)L_{r}$$ (9)

where *α* is a hyperparameter that balances tensor gradient descent between $L_{b}$ and $L_{r}$ . The procedure of BiHo-GNN is illustrated in Figure 2.

## 3 Results and discussions

### 3.1 Performance measures

In this paper, we use recall, precision, F1-Score, AUC and AUPR to evaluate the performance of BiHo-GNN. Measurements are defined as follows:

$$Recall=\frac{TP}{TP+FN}$$ (10)

$$Precision=\frac{TP}{TP+FP}$$ (11)

$$F1-Score=\frac{2\times Precision\times Recall}{Precision+Recall}$$ (12)

where TP, TN, FP, and FN denote the number of true positives, true negatives, false positives, and false negatives in the binary classification, respectively. Area under receiver operating characteristic curve (AUC) is utilized to measure the performance of the classifier with TP ratio and FP ratio. Area Under the Precision-Recall curve (AUPR) is used to evaluate the model with precision and recall.

### 3.2 Comparison with existing methods

We compare the proposed BiHo-GNN framework with five methods, including LPIGAC (Jin et al., 2021), LncPNet (Zhao et al., 2022), RWR (Random Walk with Restart) (Wiggins et al., 2016) and LPBNI (Ge et al., 2016) on NPInter2.0 (5:5). Table 2 demonstrates the performances of BiHo-GNN and the above methods.

**TABLE 2:** Performance comparison of lncRNA-protein interaction prediction.

Method	AUC	AUPR	Recall	Precision	F1-score
BiHo-GNN	**0.950**	0.899	**0.919**	0.886	0.902
LncPNet	0.938	**0.957**	0.881	**0.948**	**0.913**
LPIGAC	0.936	0.822	0.669	0.832	0.742
LPISKF	0.909	0.685	0.623	0.643	0.633
RWR	0.826	0.581	0.566	0.535	0.550
LPBNI	0.852	0.624	0.634	0.533	0.579

Bold values are the best performance of each task.

**FIGURE 3:**
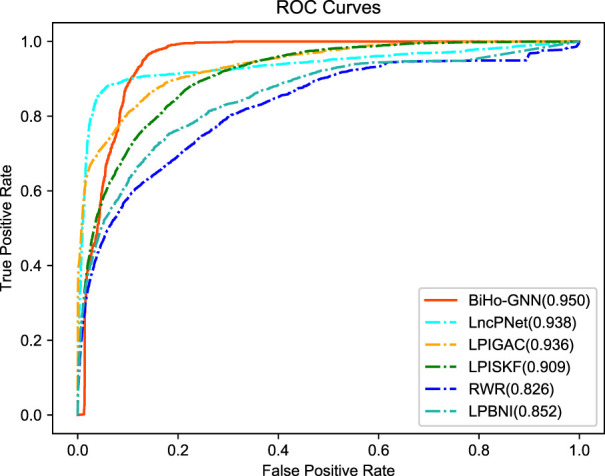
The receiver operating characteristic curve for five methods.

**FIGURE 4:**
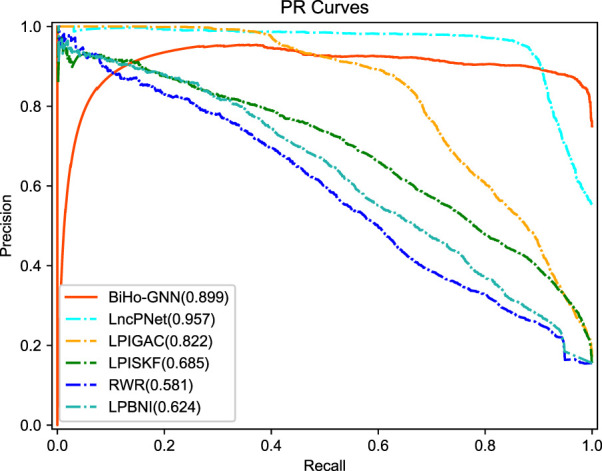
The precision-recall curve for five methods.

From the table, BiHo-GNN achieves AUC of 95.0%, AUPR of 89.9%, Recall of 0.919%, Precision of 88.6%, and F1-score of 0.902. BiHo-GNN outperforms the other five methods under the same experimental conditions, In particular, the AUC and Recall values of BiHo-GNN are increased by 1.2% and 3.8% when compared with the highest evaluation indicators among the methods. ROC curves and PR curves for BiHo-GNN are illustrated on Figures 3, 4.

In Table 2, compared with methods, BiHo-GNN achieves robust performance.

### 3.3 Comparison with multiple classifiers

The classifier of the model plays a crucial role in the evaluation of the model. We conduct experiments on different classifiers in four data sets, we compare the SVM (Wang and Hu, 2005) classifier based on the linear kernel, the classic classifier XGBoost (Li et al., 2008), the gradient boosting decision tree (GBDT) (Ye et al., 2009), the random forest classifier based on bagging (Breiman, 2001), the k-nearest neighbor algorithm (KNN) (Sun and Huang, 2010) and the logistic regression (LR) method (Kleinbaum et al., 2002). Finally, we set LR as the final classifier. Table 3 indicates that the LR classifier outperforms the other six classifiers.

**TABLE 3:** Performance comparison of different classifiers.

Datasets	Classifier	AUC	AUPR	Recall	Precision	F1-score
NPInter2.0 (5:5)	SVM	0.902	0.840	**0.979**	0.847	0.908
	XGBoost	0.640	0.637	0.285	**0.980**	0.442
	GBDT	0.556	0.553	0.117	0.957	0.209
	Random forest	0.937	**0.906**	0.946	0.930	**0.938**
	KNN	0.935	0.898	0.963	0.913	0.937
	LR	**0.950**	0.899	0.919	0.886	0.902
NPInter2.0 (4:6)	SVM	0.879	0.814	**0.958**	0.827	0.887
	XGBoost	0.655	0.652	0.316	**0.981**	0.479
	GBDT	0.581	0.577	0.174	0.941	0.294
	Random forest	0.928	**0.894**	0.935	0.922	0.928
	KNN	0.931	0.862	0.955	0.911	**0.932**
	LR	**0.944**	0.893	0.920	0.901	0.899
NPInter3.0_H (5:5)	SVM	0.816	0.763	**0.792**	0.831	**0.811**
	XGBoost	0.640	0.637	0.285	**0.980**	0.442
	GBDT	0.642	0.617	0.246	0.976	0.394
	Random forest	0.830	0.808	0.710	0.934	0.807
	KNN	0.803	0.772	0.698	0.889	0.782
	LR	**0.913**	**0.909**	0.587	0.911	0.714
NPInter3.0_H (4:6)	SVM	0.829	0.807	0.709	0.933	0.805
	XGBoost	0.655	0.654	0.314	0.990	0.442
	GBDT	0.619	0.604	0.240	**0.996**	0.386
	Random forest	0.872	0.855	0.781	0.954	**0.859**
	KNN	0.855	0.825	**0.793**	0.916	0.843
	LR	**0.923**	**0.935**	0.682	0.927	0.786
NPInter3.0_M (5:5)	SVM	0.825	0.741	**0.997**	0.741	0.850
	XGBoost	0.652	0.652	0.305	0.995	0.468
	GBDT	0.617	0.605	0.226	**0.996**	0.352
	Random forest	0.904	**0.887**	0.872	0.932	0.901
	KNN	**0.915**	0.876	0.924	0.907	**0.915**
	LR	0.866	0.791	0.945	0.747	0.835
NPInter3.0_M (4:6)	SVM	0.733	0.653	**0.976**	0.656	0.785
	XGBoost	0.644	0.639	0.300	0.962	0.458
	GBDT	0.603	0.582	0.218	**0.994**	0.327
	Random forest	0.870	**0.837**	0.820	0.911	0.863
	KNN	**0.883**	0.835	0.895	0.875	**0.884**
	LR	0.803	0.679	0.741	0.709	0.725
RPI2241 (5:5)	SVM	0.630	0.576	0.908	0.584	0.711
	XGBoost	0.606	0.601	0.212	**0.991**	0.350
	GBDT	0.616	0.589	0.195	0.982	0.356
	Random forest	**0.937**	**0.893**	**0.985**	0.898	**0.940**
	KNN	0.725	0.668	0.679	0.748	0.711
	LR	0.757	0.657	0.634	0.695	0.663
RPI2241 (4:6)	SVM	0.641	0.582	0.912	0.615	0.735
	XGBoost	0.625	0.613	0.241	**0.971**	0.416
	GBDT	0.626	0.612	0.253	0.964	0.375
	Random forest	**0.914**	**0.905**	**0.981**	0.892	**0.958**
	KNN	0.752	0.647	0.684	0.751	0.815
	LR	0.775	0.689	0.684	0.705	0.694

Bold values are the best performance of each task.

### 3.4 Performance analysis

Different from the general validation method of deep learning such as n-fold cross-validation according to the experimental parameters of the previous work (Gao et al., 2020), we split four lncRNA-protein interaction datasets into the 5:5 and 4:6, which denote the ratios of the training set and test set. This specific data set division rule limits the application of BiHo-GNN to datasets with a small amount of interactions. The performance of BiHo-GNN on four datasets is listed in Table 4.

**TABLE 4:** Performance of BiHo-GNN on four different datasets.

Datasets	AUC	AUPR	Recall	Precision	F1-score
NPInter2.0 (5:5)	0.950	0.899	0.919	0.886	0.902
NPInter2.0 (4:6)	0.944	0.893	0.920	0.901	0.899
NPInter3.0_H (5:5)	0.913	0.909	0.587	0.911	0.714
NPInter3.0_H (4:6)	0.923	0.935	0.682	0.927	0.786
NPInter3.0_M (5:5)	0.866	0.791	0.945	0.747	0.835
NPInter3.0_M (4:6)	0.803	0.679	0.741	0.709	0.725
RPI2241 (5:5)	0.757	0.657	0.634	0.695	0.663
RPI2241 (4:6)	0.775	0.689	0.684	0.705	0.694

**FIGURE 5:**
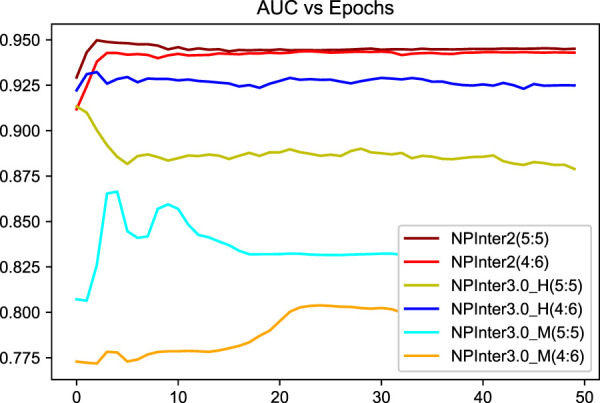
Algorithm convergence of the model under different data sets.

**FIGURE 6:**
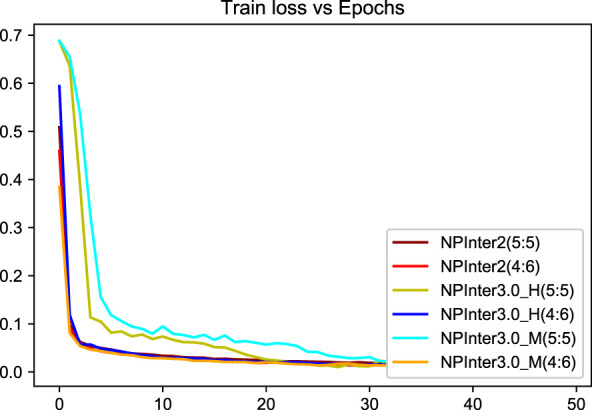
Model loss curve.

We applied unique data partitioning methods that differed from traditional deep learning validation methods such as 10-fold cross-validation, because of the particularity of the heterogeneous graph, the training set and test set are not allowed to have a big difference in the amount of data, we divided each data set into 5:5 and 4:6. Algorithm convergence of the model under different data sets and partitions are shown in Figures 5, 6.

### 3.5 Parameter sensitivity

We randomly shuffled the dataset and validated BiHo-GNN on NPInter2.0 (5:5) to train BiHo-GNN for 50 epochs on each dataset. All training processes are run on Windows 11 operation system, a single NVIDIA GeForce RTX3060 GPU with 8 GB memory and Intel(R) Core(TM) i3-12100F CPU @ 3.30 GHz. The hyper-parameter in the model has an impact on the performance of the model. As shown in Table 5, the harmonic factor *α* ∈ {0.1, 0.3, 0.5, 0.7, 0.9} with step length 0.2 is selected for investigating the lncRNA-protein relationship. When *α* = 0.9, our framework achieves the best performance on NPInter2.0 (5:5) and NPInter2.0 (4:6).

**TABLE 5:** Performance and standard deviation with five harmonic factor *α*.

Datasets	*α*	AUC	AUPR
NPInter2.0 (5:5)	0.1	0.9218 ± 0.0037	0.8152 ± 0.0041
	0.3	0.9347 ± 0.0038	0.8451 ± 0.0034
	0.5	0.9431 ± 0.0062	0.8652 ± 0.0045
	0.7	0.9429 ± 0.0043	0.8632 ± 0.0037
	0.9	**0.9501 ± 0.0046**	**0.8994 ± 0.0025**
NPInter2.0 (4:6)	0.1	0.9164 ± 0.0055	0.8027 ± 0.0043
	0.3	0.9244 ± 0.0126	0.8371 ± 0.0152
	0.5	0.9348 ± 0.0036	0.8582 ± 0.0046
	0.7	0.9326 ± 0.0052	0.8569 ± 0.0028
	0.9	**0.9442 ± 0.0042**	**0.8933 ± 0.0058**

Bold values are the best performance of each task.

We implement BiHo-GNN with packages PyTorch 1.11.0 and PyTorch-geometric. Adam optimizer is adopted for gradient optimization. According to the scale of the dataset and the computation complexity of our framework, we set the feature dimension of the node to be 128. The learning rate is selected from {0.001, 0.005, 0.01, 0.05, 0.1} to evaluate the model performance, and verification results show that the proper learning rate is 0.001. Moreover, the learning rate decay is the learning rate multiplied by a hyper-parameter. When the training loss rises, the learning rate decay is set to 0.9, and the results with NPInter2.0 (5:5) and NPInter2.0 (4:6) are shown in Table 6.

**TABLE 6:** Performance and standard deviation with four different learning rate.

Datasets	lr	AUC	AUPR
NPInter2.0 (5:5)	0.001	0.9357 ± 0.0039	0.8732 ± 0.0037
	0.01	**0.9501 ± 0.0046**	**0.8994 ± 0.0025**
	0.05	0.9285 ± 0.0024	0.8648 ± 0.0035
	0.1	0.9159 ± 0.0072	0.8541 ± 0.0083
NPInter2.0 (4:6)	0.001	0.9235 ± 0.0051	0.8528 ± 0.0024
	0.01	**0.9442 ± 0.0042**	**0.8933 ± 0.0058**
	0.05	0.9157 ± 0.0036	0.8426 ± 0.0024
	0.1	0.9014 ± 0.0039	0.8274 ± 0.0072

Bold values are the best performance of each task.

Moreover, we evaluate different hidden dimensions of neural networks on BiHo-GNN. As shown in Table 7, when the hidden dimension at 128, BiHo-GNN achieves the best performance on NPInter2.0 (5:5) and NPInter2.0 (4:6), we set the hidden dimension to 128 to balance the cost of time and space of the model.

**TABLE 7:** Performance and standard deviation with seven different hidden dimensions.

Datasets	Hidden dimensions	AUC	AUPR
NPInter2.0 (5:5)	49	0.9325 ± 0.0057	0.8621 ± 0.0027
	64	0.9356 ± 0.0083	0.8694 ± 0.0138
	81	0.9423 ± 0.0034	0.8792 ± 0.0045
	100	0.9449 ± 0.0057	0.8649 ± 0.0084
	128	**0.9501 ± 0.0046**	**0.8934 ± 0.0025**
	144	0.9258 ± 0.0075	0.8493 ± 0.0064
	169	0.9136 ± 0.0063	0.8346 ± 0.0039
NPInter2.0 (4:6)	49	0.9285 ± 0.0047	0.8635 ± 0.0068
	64	0.9263 ± 0.0043	0.8644 ± 0.0118
	81	0.9385 ± 0.0085	0.8726 ± 0.0036
	100	0.9358 ± 0.0038	0.8685 ± 0.0079
	128	**0.9442 ± 0.0042**	**0.8933 ± 0.0058**
	144	0.9155 ± 0.0039	0.8495 ± 0.0075
	169	0.9025 ± 0.0085	0.8329 ± 0.0058

Bold values are the best performance of each task.

### 3.6 Case study

In this section, we mask lncRNA-protein interaction in database NPInter2.0 (Yuan et al., 2014) to infer possible potential associations and verify our result by publications. The predicted top 10 lncRNA-protein interaction is described in Table 8. From the table, we can observe EWSR1 is interacting with NONHSAG029787, NONHSAG008595, and NONHSAG008586, which is associated with non-small-cell Lung, lymphoma, and malignant glioma diseases (Paronetto et al., 2014). NONMMUG000162, NONHSAG055885, NONMMUG038556, and NONMMUG039105 can affect the transcription of the protein AGO4. Urinary bladder neoplasms, uterine cervical neoplasms, and thyroid cancer can be activated by the above interaction pairs (Yuan et al., 2014).

**TABLE 8:** The predicted top 10 potential lncRNA-protein interaction pairs with BiHo-GNN.

LncRNA	Protein	Comfirmed	PubMed
NONHSAG029787	EWSR1	Yes	24813895
NONHSAG008595	EWSR1	Yes	22955616
NONHSAG008586	EWSR1	Yes	22955616
NONMMUG000162	AGO4	Yes	29167373
NONHSAG055885	AGO4	No	—
NONMMUG038556	AGO4	Yes	29167373
NONMMUG039105	AGO4	Yes	29167373
NONHSAG008584	EWSR1	Yes	22955616
NONHSAG008517	EWSR1	Yes	22955616
NONHSAG008516	EWSR1	Yes	22955616

## 4 Conclusion

LncRNAs are responsible for the regulation of many critical biological processes, such as protein transcription. These two molecular interaction information are closely related to multiple human diseases. It is a significant work to predict potential lncRNA-protein interaction and to study heterogeneous network learning.

In this paper, we propose the novel framework BiHo-GNN for predicting lncRNA-protein interaction. BiHo-GNN utilized bipartite embedding generated by Bi-GNN Encoder. Our work first integrates bipartite graph neural networks and homogeneous graph networks, which strongly verifies the feasibility of heterogeneous graph networks in predicting lncRNA-protein interaction and similar link prediction problems.

Model performance comparison and case study show that BiHo-GNN outperforms state-of-the-art methods on all selected datasets in this paper. Compared with other models using bipartite graph features, BiHo-GNN can well integrate the features of homogeneous networks and heterogeneous networks.

## Data Availability

Publicly available datasets were analyzed in this study. This data can be found here: Publicly available datasets were analyzed in this study. NPInter2.0 database can be found https://github.com/zhanglabNKU/BiHo-GNN/tree/main/BiHo/dataset_preprocessing/dataset, NPInter3.0 database can be found http://bigdata.ibp.ac.cn/npinter4/download/, RPI2241 database can be found https://github.com/zhanglabNKU/BiHo-GNN/tree/main/BiHo/dataset_preprocessing/dataset. Full codes of the BiHo-GNN project are available at our GitHub repository https://github.com/zhanglabNKU/BiHo-GNN.
